# Positive Environments and Precautionary Behaviors During the COVID-19 Outbreak

**DOI:** 10.3389/fpsyg.2021.624155

**Published:** 2021-03-11

**Authors:** Víctor Corral-Verdugo, Nadia S. Corral-Frías, Martha Frías-Armenta, Marc Yancy Lucas, Edgar F. Peña-Torres

**Affiliations:** ^1^Department of Psychology, Universidad de Sonora, Hermosillo, Mexico; ^2^Department of Human Nutrition, Universidad Estatal de Sonora, Hermosillo, Mexico

**Keywords:** positive environment, resources, challenges, precautionary, COVID-19, wellbeing

## Abstract

Theoretically, a positive environment (PE) includes (a) tangible and intangible resources that satisfy human needs, (b) enablers of healthy, pro-social, and pro-environmental behaviors that guarantee socio-environmental quality and wellbeing, and (c) environmental challenges that must be faced and solved. One of the most salient challenges is the global COVID-19 pandemic. This study sought to investigate whether PEs can stimulate responsible actions (i.e., self-care and precautionary behaviors against COVID-19), while maintaining personal wellbeing. Nine hundred and forty-nine Mexicans participated in an online survey encompassing five primary factors: resources, enablers, challenges, responsible health behaviors, and wellbeing. The first three factors examine “resources” such as physical infrastructure as well as family and social support, “enablers” which include information about protective health practices and perceived legitimacy of authorities in handling the pandemic, and “challenges” encompassing threat perception and social pressure to not engage in precautionary measures. Participants also self-reported hedonic wellbeing as well as self-care and precautionary behaviors, which formed the “responsible (health) behavior” factor. Structural equations model (*n* = 714 after list-wise deletion) showed that “resources,” “challenges,” and “enablers” form a second-order factor, “positive environments,” and this factor strongly covaries with “responsible behavior” and “wellbeing.” These results suggest that PEs are not only buffers against the negative impact of the COVID-19 pandemic but can also stimulate effective responses against a threat while maintaining individual wellbeing. These results can be used to inform the development and maintenance of PE frameworks aimed at minimizing the spread of COVID-19 and encouraging mental and physical health.

## Introduction

The COVID-19 pandemic constitutes one of the most pressing challenges worldwide currently. Quarantine, social/physical distancing, and self-isolation represent effective frontline precautionary behaviors that reduce viral transmission. These actions invite new questions about sociophysical space and the role it plays in public health and safety. Despite the protective effects on physical health, other potential negative outcomes (i.e., depression, obesity, and feelings of isolation) may affect the lives of billions of people worldwide. As such, a better understanding of the sociophysical factors that influence safe, precautionary behavior is critical for an effective response moving forward. This paper proposes that positive environments (PEs) (i.e., settings that satisfy human needs, promote responsible behaviors and provide challenges) may encourage individual precautionary response toward pandemics like COVID-19.

The COVID-19 pandemic, caused by the SARS-CoV-2 virus, continues to remain a serious global health emergency, particularly in the Americas. The Pan-American Health Organization (*Organización Panamericana de Salud, OPS*) estimated in August 2020 that while the region accounts for approximately 13% of the global population, it has accounted for around 64% of the reported deaths (United Nations Organization, 2020). As of October, the United States, Brazil, and Mexico, represented the countries with the first, second, and fourth highest total reported deaths worldwide. Combined these three nations accounted for over 440,000 deaths (41% of total deaths worldwide). Mexico has experienced a particularly high rate of death as a percentage of confirmed infected population at 10% (Johns Hopkins Csse, 2020). Furthermore, it is estimated that COVID-19 will be the number one cause of death in Mexico by the end of 2020 (IHME, 2020). Perhaps more concerning is the likelihood that these are underreported figures and that the full extent of the infection and related death is even greater (Lau et al., 2020).

Beyond the catastrophic direct negative effects of the virus, other indirect mental and physical health concerns like depression, anxiety, and family violence appear to be increasing. The director of the OPS has claimed the Pan-American region is experiencing a “perfect storm” of events leading to an unprecedented mental health crisis. Uncertainty, stress, and stay-at-home orders may have exacerbated these issues that likewise may be underreported in the wake of reduced access to support and extension services (United Nations Organization, 2020). In a sample of Mexican participants taken 1 week after the announcement of a national emergency, 50% reported their *psychological distress* as moderate to severe in the context of a specified event (that may be interpreted by respondents as the COVID-19 pandemic). Female participants reported higher rates across measurements (i.e., anxiety, depression, and stress), while respondents that live with at least one other individual reported higher rates of anxiety and psychological distress (Cortés-Álvarez et al., 2020).

Viral transmission, to the best of our current understanding, is primarily spread *via* personal contact and respiratory droplets (*via* coughing, sneezing, talking, singing, etc.). Airborne transmission over farther distances may be an issue as well, particularly in locations with poor ventilation (Jayaweera et al., 2020; Park et al., 2020; World Health Organization, 2020). It appears that while fomite transmission (from inanimate objects or surfaces) may pose some risk, the likelihood of acquiring the virus in this manner *via* real-life scenarios appears low (Goldman, 2020). While critical research is still needed to understand the specifics how SARS-CoV-2 (the cause of COVID-19) transfers from person-to-person, human contact appears to be the frontline of virus transmission. In the absence of a vaccine, the best-known way for individuals to prevent virus transmission is *precautionary behavior* (i.e., physical distancing, wearing masks, washing hands, surface cleaning, and avoiding touching the eyes, nose, or mouth) (Lee and You, 2020). Precautionary behaviors may also include isolation of individuals with confirmed cases or those showing COVID-19 like symptoms as well as a temporary cessation of educational, cultural, and economic activities. However, while these measures appear to reduce the chances of viral transmission, they are not without potential negative socio-psychological effects (including depression, stress, anxiety, sleep alteration, social stigma, loss of income, and disruption of normal social life) (Pfefferbaum and North, 2020). As such, a comprehensive understanding of the factors that promote precautionary behaviors without compromising individual wellbeing can inform future policy initiatives and guide mitigation efforts moving forward.

### Positive Environments

Evidence has suggested that a PE represents a sociophysical setting that allows people to cope with emergencies, crises, and environmental challenges. A PE is conceived as “a context that promotes individual and collective benefits and that also influences human predispositions to conserve—in the long run—the sociophysical structures on which life depends” (Corral-Verdugo and Frías-Armenta, 2016, p. 965). PEs allow people to flourish and experience physical and mental wellbeing, while mitigating the negative consequences of stressful, painful, or unpleasant conditions (Valera and Vidal, 2017). In a PE, people experience personal and social growth; personal growth refers to the process of becoming better in a personally meaningful way (Vittersø, 2014), while social growth implies the development of an individual’s knowledge and ability in dealing with other individuals and groups (APA, 2021).

The construct also considers the satisfaction of psychosocial needs such as affiliation with others and affection and social support from family, friends, and peers (Deci and Ryan, 2000).

In addition, PEs enable socially and environmentally responsible behaviors that can also be understood as sustainable (Corral-Verdugo and Frías-Armenta, 2016). Therefore, a positive setting provides resources that enable the individual to successfully face environmental challenges while simultaneously requiring the conservation of those resources for future needs.

*Resources* provided by the environment (setting), tangible (material) or intangible (psychosocial), represent core elements of PEs. Evidence has demonstrated that physical environmental resources are crucial components for an effective health crisis response. For instance, access to safe public open space (e.g., exposure to blue-green spaces, rooftops, trails that allow for adequate physical distancing) has demonstrated beneficial effects for mental health (Bratman et al., 2019). Recent studies in pandemic response have suggested that contact with nature may buffer the effects of COVID-19 related stress. In a study of Italian participants, access to views of green areas were associated with a reduction in self-report depression symptoms (Amerio et al., 2020). Similarly, a pre-print study canvassing 77 European countries (61% Spanish sample) found that individuals who maintained contact with natural environments, either through access to private outdoor spaces or blue-green natural viewsheds, reported fewer depression and anxiety symptoms as well as greater self-report positive mood (Pouso et al., 2020).

Family and social support factors represent another critical resource of PEs that influence how individuals react to major events like the COVID-19 pandemic. Positive family functioning, including family support, has demonstrated a relationship with reduced illness incidence and greater adoption of general health practices (García-Huidobro et al., 2012). Similarly, social support and peer influence have shown to be determinants of precautionary behaviors (Hurdle, 2001). The influence of family and social support appears to be reinforced during health crises, representing an important buffer against the negative effects of contagious outbreaks. In a study of Hong Kong residents during the SARS epidemic of 2003, participants reported greater concern for the feelings of family members and friends (over 60%) and about two-thirds stated that they paid more attention to their mental health than they had 2 months prior. Participants likewise reported greater social support from friends (28% increase) and family members (39%) when in need as well as higher rates of sharing feelings with family (35%) related to their experience 2 months before. In general, these factors were negatively associated with post-traumatic stress, stress perception, and other SARS-related perceptions (Lau et al., 2006). Recent studies suggest the influence of social and familial support may be greater in the wake of a more severe and widespread public health event like COVID-19. A study of the Liaoning Province in mainland China, which used the same items as the previous research, undertaken during the early stages of official response (28th January through 5th February 2020), found participants reported greater support from friends (65%) and family members (64%) and that most (58%) reported increased shared feelings among family members compared to 2 months prior. Furthermore, nearly 78% reported increased care for the feelings of family members (Zhang and Ma, 2020).

Furthermore, in a PE, the responsible actions of individuals are triggered by affordances that enable pro-environmental and prosocial behaviors making possible the conservation and integrity of that environment. These *enablers* of responsible actions include physical urban design that facilitates sustainable actions, information to cope/solve environmental problems, social models of responsible behavior, perceived legitimacy of authorities tasked with addressing important social issues, and government programs guaranteeing access to social justice and equity, (Corral-Verdugo et al., 2017). It is possible to identify enablers of responsible behaviors (i.e., healthy, precautionary) in the framework of COVID-19. Previous research has demonstrated that enablers, like access to relevant information suggesting guidelines for pandemic response (Lau et al., 2003) and perceived legitimacy of authority in managing the health crisis (Hartley and Jarvis, 2020), can help guide official response and encourage public compliance with offered suggestions.

Individual behavior can be triggered by the presence of *environmental challenges* (Corral-Verdugo et al., 2015b). These challenges may be environmental threats, extreme environments, personal adversity, illness, and social problems. If those challenges combine with the presence of personal capacity and social resources, they can encourage personal and social growth. A trilogy working in sequence (challenges recruit personal capacities and resources which in turn lead to problem solving) manifests in these cases, producing positive outcomes (Suedfeld, 2012). Environmental challenges may serve as catalyzers of pro-environmental reflection and action. Experiencing a crisis prompts individuals to internalize and take action to ameliorate environmental problems (McDonald-Harker et al., 2020). Problem-solving that results from effectively acting in response to environmental challenges is identified as adaptive actions, competency display, and societal development, within the framework of a PE (Corral-Verdugo et al., 2017). The threat represented by COVID-19, undoubtedly, constitutes a formidable challenge for people around the world, even if most people–especially the younger–do not believe they will contract a disease caused by a novel virus (Commodari and La Rosa, 2020; Commodari et al., 2020). Although risk perception of public health crises may result in depression (Ding et al., 2020), threat perception may also lead to compliance of public health recommendations (Barr et al., 2008), and social pressure also affecting compliance with those recommendations (Hurdle, 2001) are instances of environmental challenges in the context of COVID-19. Social pressure is a challenge since it tests the ability or determination of an individual to engage in a behavior. In this case, compliance with precautionary measures. This means that, in addition to be facing the pandemics, people must cope with pressures to not practicing actions preventing them from getting the disease.

These three factors (resources, challenges, and enablers) are the constitutive elements of a PE. The PE that results from their combination influences (and is influenced by) the sustainable behaviors of individuals and, likewise of equal importance, personal wellbeing. These bidirectional influences imply that, in a PE, individuals engage in responsible behaviors and experience wellbeing creating a recursive chain of positive interdependences between settings, behaviors, and wellbeing (Corral-Verdugo et al., 2015b).

*Responsible (sustainable) behaviors* are activities that include pro-environmental and pro-social actions (Tapia-Fonllem et al., 2013). Self-care behaviors are also part of the set of sustainable/responsible behaviors (Corral-Verdugo et al., in press). Self-care consists of a series of actions adopted by individuals to seek personal wellbeing in the physical, mental, spiritual, and intellectual dimensions (Tobón Correa, 2003). Pro-social, pro-environmental and self-care behaviors are significantly interrelated (Corral-Verdugo et al., in press). An individual’s health not only depends on personal care, but also on the social system determining the relations between self-care and the protection of the socio-physical environment (Orem, 1993). Pertinent to our study, self-care is related to precautionary behaviors, that is self-protective actions facilitate the practice of precautionary behaviors (Márquez-Serrano et al., 2012), suggesting that those who care for themselves also tend to protect others and their environment. Although, there is some overlapping between self-care and precautionary behavior, they differ in the level of specificity; whereas self-care involves actions aimed at maintaining general health (in both physical and mental matters), precautionary behaviors are directed to preventing the infection from the SARS-CoV-2 virus.

*Wellbeing* is experienced in PEs and is associated with responsible behaviors. Responsible behavior is not, of course, the sole cause of wellbeing, yet in situations where high quality of life and satisfaction exists (i.e., wellbeing), social support, and physical resources (i.e., PE resources) are more likely to be present (Van den Berg et al., 2007; Semenza and March, 2009; Schulte and Vainio, 2010). In addition, the practice of sustainable behaviors has a bidirectional relationship with human wellbeing (Prati et al., 2017). As such, the practice of environmentally and socially responsible behaviors result in higher levels of wellbeing but, simultaneously, the experience of wellbeing stimulates engagement in responsible behaviors. Regarding health care, positive affect and hedonic wellbeing are related to personal care (Kessing et al., 2014). Conversely, anhedonia predicts important clinic events (Denollet et al., 2008) and deficiencies in pleasure are considered to constitute an important affective mechanism impacting self-care (Leventhal, 2012).

**FIGURE 1:**
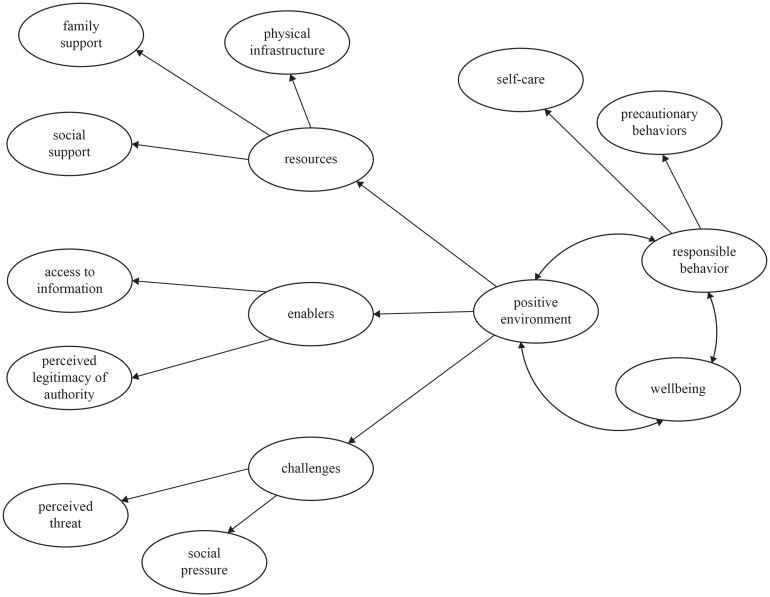
Theoretical model of positive environments and precautionary behaviors against COVID-19.

Figure 1 represents the proposed chain of events that link a PE, and its elements, in responsible (sustainable) behaviors and human wellbeing in the context of a pandemic.

Considering the theoretical and antecedent research, the present study was designed to test a model of PEs in the context of the COVID-19 pandemic. According to this model, a PE, formed by physical, family, and social *resources*, as well as environmental *challenges* (threat perception, social pressure), and environmental *enablers* (information, legitimacy of authorities), should influence and be influenced by responsible (self-care/precautionary) behaviors against COVID-19 as well as influence personal wellbeing. For the PE construct, the theory establishes that resources, enablers, and challenges are factors that *should* be all present, and correlate with each other to produce an optimal level of positivity, and both the expected (responsible) behaviors and wellbeing. In this sense, PE is assumed to be a unitary and coherent construct representing a conjunction of favorable environmental conditions: The interrelation between these conditions is required to produce those effects. Yet, a certain level of positivity may exist in the environment with the presence of two (enablers, resources, for example) or even one (resources, for instance) elements, and the contribution of every indicator to positivity does not depend on the level of contribution of other indicator(s).

Therefore, according to this model, the elements of a PE should interrelate significantly and, in turn, will be associated with responsible behaviors and wellbeing. This predicts that, in a PE, people tend to practice healthy and precautionary behaviors and, simultaneously, experience higher levels of wellbeing.

## Materials and Methods

### Participants

Data was collected using a snowball recruitment procedure. Invitations to participate were sent *via* email, text, and social media in keeping with physical distancing guidelines. All participants were informed of the aims, benefits, and study risks before signing a digital consent form prior to participating. The sample included 949 individuals from all the 32 Mexican states. All participants above the legal age of consent in Mexico (18 years) were eligible. Mean age was 40.89 (SD = 14.77), ranging from 18 to 78. Most participants, (501) self-identified as female, 227 identified as males, five as non-binary and 13 preferred not to answer. Approximately, one-third of the participants reported being married (34.9%) and a quarter of the sample reported being single (24.8%), remaining participants reported being either divorced, widowed, or living in cohabitation (5.7, 1.9, and 11.6% respectively) with 21.2% choosing not to respond. Approximately, more than a third of participants completed college degree (35.7%), and about a fourth reported completing a postgraduate degree (24.6%). Less than 2% reported completing elementary or middle school (0.1 and 1.7% respectively) and 14.8% reported completing high school. On a 1–10 subjective social ladder scale (based on MacArthur ladder), 39.5% of respondents classified themselves as having a mid-level social status (5–7 interval), 13.6% self-defined as lower status (1–4 intervals) and 10.6% self-classified as higher status (8–10 interval); 24.2% did not respond.

### Procedure

The questionnaire was distributed between the end of May and the beginning of June 2020, 3 months after the pandemic started in Mexico. During this time, health and government officials continued to issue a “stay at home” request (#quedateencasa), which in most states was mandatory. Likewise, health and government officials disseminated informational campaigns about COVID-19 and hygienic measures to avoid contracting and spreading the virus.

Academic groups from various Mexican educational and research institutions were contacted electronically and invited to participate in the study. Academics were asked to subsequently distribute the invitation within their network. A Facebook page was also created to recruit participants. Data was collected using Qualtrics software. Participants were informed of the study aims and were informed that they could stop responding at any time if they decided to do so. Approximately 2% of those who received the link declined to participate. All the procedures used in this study comply with the ethical standards of national and international human-subjects committees and were approved by the University of Sonora Ethics Committee.

### Instruments

We used different psychosocial instruments to test the PEs model during COVID-19. A positive COVID-related environment was assessed using family support, social support, perception of legitimate authorities, social pressure to break quarantine, and perception of access to COVID-safe public outdoor areas. Other psychological variables included threat perception and hedonic wellbeing. We evaluated behavioral variables associated to general health practices (i.e., self-care) and pandemic-specific precautionary behavior against COVID-19. The questionnaire also included socioeconomic items such as gender, age, education, and occupation. All instruments were either created or adapted specifically for this study. Several scales were reduced to reduce participant response time.

#### General Health Practices

General health practices (state health practices) were assessed using five items from a self-care instrument (Corral-Verdugo et al., in press), and one item addressing general health. Questions addressed self-care practices such as exercise and eating habits, where a five-point Likert-type scale ranging from “never” (1) to “always” (5) was used. This reduced version of the scale showed acceptable internal consistency in a similar Mexican sample (α = 0.64) (Frías-Armenta et al., in press).

#### Hedonic Wellbeing

The six reversed-key items in the anhedonic depression subscale from the Mini-MASQ (Casillas and Clark, 2000) scale were utilized to assess hedonic wellbeing. Participants responded to items like “I feel happy” and “I feel that I have a lot of things to do” using a 5-point Likert-type scale of agreement (1 “nothing” to 5 “extremely”). The Mini-MASQ has been previously validated in Mexico (Corral-Frías et al., 2019) and the 8-item anhedonic depression subscale reported acceptable internal consistency (α = 0.83).

#### Precautionary Behaviors

Precautionary measures against COVID-19 items were adapted from a previous study (Frías-Armenta et al., in press). It included seven Likert-type questions assessing COVID-19-related preventive behaviors taken by participants 3 days prior to their participation. The items included how often people try to avoid touching their eyes, nose, and mouth when out in public as well as hand-washing behaviors, use of sanitizing disinfectants to clean items before they come into the home, adherence to stay at home orders, proper sanity behaviors when sneezing/coughing, mask usage, and maintenance of a 6-foot minimum physical distance.

#### Legitimacy

This scale was written specifically for this study, based on Tyler (2006) approach to Legitimacy expressed as the support, trust, and confidence in legal authorities. Six items were elaborated but only three were used to improve the model’s fit indices. Participants answered using a 5-point Likert-type scale, to items related to their confidence that legal authorities are considering and recommending the best decisions regarding COVID-19, looking out for the best intentions of the public, and acceptance of official decisions. Internal consistency between the three items was acceptable (α = 0.83).

#### COVID-19 Information

To assess individual information on COVID-19, three 5-point Likert-type (0 = never, 1 = almost = never, 2 = once during the week, 3 = every day, and 4 = more than once in the day) items were constructed specifically for this study. Items asked about the frequency of individual research regarding COVID-19 as well as the manners used to search for information, health measures adopted to prevent the spread of COVID-19, and new health recommendations. Internal consistency between these four items was acceptable (α = 0.75).

#### Social Pressure to Break COVID-19 Social Distancing

To assess social pressure to break social distancing a scale was specially constructed for this study. The items were formatted using a 5-point Likert-type scale (0 = strongly disagree, 1 = disagree, 2 = neutral, 3 = agree, and 4 = strongly agree). Items included statements such as, my friends keep going out and taking part in social gatherings, my friends insist on continuing to take part in social meetings, my friends and family continue lead a normal life unincumbered by precautionary behaviors, and my acquaintances do not believe COVID-19 is dangerous. The internal consistency between these three items was acceptable (α = 0.75).

#### Threat Perception

To assess threat perception of COVID-19, a translated and modified version of the Perceived Coronavirus Threat Questionnaire was used (Conway et al., 2020). The final instrument consisted of five 5-point Likert-style items. The “I am worried that I or people I love will get sick from the corona virus (COVID-19)” was eliminated. The original six-item translation was validated in student sample and showed good internal consistency (α = 80). The five items used here also showed good internal consistency (α = 80).

#### Family and Social Support

To assess family and social support an adaptation of the support networks instrument from Villalobos (2009) was used. The instrument elicits responses on the degree to which participants feel supported by family, friends, or other persons and institutions. For this study, eight 5-point Likert-type (0 = strongly disagree, 1 = disagree, 2 = neutral, 3 = agree, and 4 = strongly agree) items were used. Some items included were “I can trust good friends” and “In my social circle, people support me.” The items showed acceptable internal consistency (α = 0.74).

#### Physical Infrastructure (Access to Safe Outdoor Spaces)

Five 5-point Likert items were constructed (1 = Strongly disagree to 5 = Strongly agree) to assess access to safe outdoor environments. Items included statements such as “I have access to an area where I can go out to do a physical activity in a safe way.” The instrument showed acceptable internal consistency (α = 0.80).

### Data Analysis and Model Specification

Internal consistency reliability (Cronbach alpha) and univariate (means and standard deviations) analyses were calculated using SPSS v.25. A structural equation model analyzing the relations between PEs, responsible behaviors and wellbeing was specified and tested using Maximum Likelihood as an estimation method. From the total 949 initial participants, 235 were excluded due to incomplete data, leaving 714 participants for data analysis. In addition, the data was randomly split in half. Then, two structural models were specified with the resulting samples to conduct an additional test of validity for our measures.

In the final model with the total sample, the unidimensionality of the instruments was tested within the measurement model. Significant (*p* < 0.05) and salient (≥0.30) factor loadings were expected, as indications of convergent construct validity for all measures. To evaluate the model’s goodness of fit, three types of fit index indicators were considered: practical, statistical, and population (Bentler, 2007). The statistical indicator was chi squared (χ^2^). To make the χ^2^ test less dependent on sample size, we used the relative χ^2^, which is calculated by dividing the χ^2^ fit index by the degrees of freedom. In accordance with previous research (Schumacker and Lomax, 2004), if this ratio is <5 the model is deemed to have good fit. Since statistical indicators are particularly sensitive to sample size, practical indicators were also considered. These included the Comparative Fit Index (*CFI*), and the Bentler–Bonnet Non-Normed Fit Index (*BBNNFI*). The *RMSEA* population fit index was also utilized.

Our model proposed that the physical infrastructure, family support, and social support factors emerge from their corresponding indicators’ (i.e., items). The common variance of these first-order factors, in turn, constitute the second-order factor “Resources.” Similarly, access to information, and perceived legitimacy of authorities formed the “Enablers” second-order factor, while perceived threat to COVID-19 and social pressure resulted in the “Challenges” second-order factor. The third-order factor “Positive Environment” emerged from the covariance of Resources, Enablers, and Challenges. In addition, “Responsible Behavior” is a second-order factor that is a result of the correlation between “Self-care” and Precautionary Behavior.” “Wellbeing” is a first-order factor assessed using items from the Hedonic Wellbeing scale. These interrelations conformed to the measurement model. The structural model indicated that the PE, Precautionary Behavior, and Wellbeing factors should highly and significantly covary.

## Results

Table 1 shows the means, standard deviations, and internal consistency coefficients (Cronbach’s alpha) for each instrument used in this study. For the Self-care and Precautionary behavior scales (part of the responsible behavior factor) the internal consistency coefficients were 0.60 and 0.65, respectively. The Challenges factor (Threat perception and Social Pressure) presented coefficients between 0.75 and 0.80. In turn, the Positive Environment factor (Physical infrastructure, Family support, Social support, Information, and Legitimacy of authorities) showed alpha coefficients between 0.71 and 0.88. Finally, the Hedonic Wellbeing factor produced an alpha coefficient of 0.85.

**TABLE 1:** Reliability and univariate statistics of scales (scale range of responses: 1–5).

**Scale/items**	**Mean**	**SD**	**Alpha**
Physical Infrastructure	**3.65**	**1.27**	**0.80**
Access for physical activity	3.57	1.28	
Access to areas to breathe fresh air	3.55	1.25	
Green area close to home to relaxe	3.49	1.35	
Patio, roof, or balcony at home	4.26	1.12	
Natural areas nearby	3.38	1.38	
Family Support	**4.15**	**0.81**	**0.71**
At home, we help each other	4.40	0.74	
At home, we treat each other with love and respect	4.39	0.74	
At home, everyone is by their side (reversed)	3.67	0.99	
Social Support	**4.05**	**0.93**	**0.88**
When in trouble, I can tell my friends	3.90	0.99	
When sad or troubled, my friends make me feel better	3.90	0.95	
I can trust good friends	4.16	0.96	
In my social circle, people support me	4.17	0.85	
I feel supported by people, besides my family and friends	4.13	0.92	
Self-care	**3.80**	**0.89**	**0.60**
Does physical activity regularly to maintain health	3.38	1.02	
Tries to consume healthy food	3.89	0.70	
Engages in activities promoting spirituality	3.06	1.19	
Rests to recover health and energy	4.07	0.79	
Does things that provide pleasure	4.02	0.74	
Tries to be in peace with her/himself	4.38	0.65	
Threat Perception	**4.47**	**1.62**	**0.80**
Thinking of coronavirus makes me feel threatened	4.23	1.58	
Afraid of the coronavirus	4.14	1.66	
Concerned about coronavirus	5.17	1.63	
Worried about catching Coronavirus	4.69	1.56	
Stressed around other people because of COVID	4.16	1.70	
Social Pressure	**2.47**	**1.09**	**0.75**
Most of my friends keep going out on the streets	2.62	1.06	
Most friends keep doing social gatherings	2.07	1.06	
Despite COVID, people continue leading their normal life	3.23	1.21	
Most acquaintances do not believe COVID is dangerous	2.46	1.08	
My friends insist that we meet	2.01	1.06	
Information	**3.40**	**0.92**	**0.75**
Information about number of cases	2.91	1.02	
Information regarding health measures against COVID	3.04	0.86	
Information about new health recommendations	4.26	0.88	
Legitimacy of Authorities	**2.89**	**1.12**	**0.83**
I am confident that authorities will make best decisions	2.84	1.15	
I am confident that authorities have the best intentions	3.04	1.14	
I would accept, without hesitation, their decisions	2.77	1.08	
Precautionary Behaviors Against COVID	**4.33**	**0.86.**	**0.65**
Avoid touching eyes, nose, and mouth without washing	3.91	1.07	
Washing hands with soap and water for at least 20 s	4.43	0.75	
Using sanitizer to clean things that come into house	4.23	1.08	
Staying at home	4.03	0.78	
Covering mouth with arm when sneeze/cough	4.68	0.60	
Wear a mask when leaving home	4.79	0.52	
Maintain a six feet minimum distance from others	4.66	0.56	
Hedonic Wellbeing	**3.23**	**0.97**	**0.85**
Felt really happy	3.32	0.90	
Felt like I was having a lot of fun	2.85	1.03	
Felt like I had a lot of energy	3.05	0.98	
Felt really “up” or lively	3.22	0.93	
Felt like I had a lot of interesting things to do	3.32	1.02	
Felt like I had a lot to look forward to	3.63	0.96	

In a 1–5 range of response, the highest means resulted from the scales Threat perception (4.47, SD = 1.62); Precautionary Behavior (4.33, SD = 0.86); Family support (4.15, SD = 0.81); and Social support (4.05, SD = 0.93). These were followed by Self-care (3.80, SD = 0.89); Physical infrastructure (3.65, SD = 1.27); Information (3.40, SD = 0.92); and Hedonic Wellbeing (3.23, SD = 0.97); which produced a moderate level of responses. The lowest means were for Legitimacy of authorities (2.89, SD = 1.12), and Social pressure (2.47, SD = 1.09).

The two structural models conducted with the randomly split data produced significant (*p* < 0.05) lambdas and structural coefficients that were similar to each other’s values and the ones found in the final structural model (see Table 2). High and significant covariances between PE, Responsible Behavior, and Hedonic Wellbeing (0.82, 0.46, and 0.73, first model;0.80, 0.42, and 0.80, second model) were obtained in these two preliminary models. The goodness of fit indicators [First model: χ^2^ = 1770.51 (1,114 df), *p* < 0.0001, *BNNFI* = 0.90, *CFI* = 0.90; *RMSEA* = 0.04; second model: χ^2^ = 1724.66 (1,114 df), *p* < 0.0001, *BNNFI* = 0.89, *CFI* = 0.90; *RMSEA* = 0.04] reveal that the models are supported by the data.

**TABLE 2:** Correspondence between lambdas of half-split samples.

	**Sample 1**	**Sample 2**

**Item**	**Lambda on factor**	**Lambda on factor**
Physical infrastructure 1	0.70	0.73
Physical infrastructure 2	0.79	0.77
Physical infrastructure 3	0.88	0.89
Physical infrastructure 4	0.24	0.33
Physical infrastructure 5	0.72	0.75
Family support 1	0.82	0.73
Family support 2	0.78	0.69
Family support 3	0.55	0.44
Social support 1	0.78	0.76
Social support 2	0.79	0.82
Social support 3	0.82	0.84
Social support 4	0.78	0.72
Social support 5	0.71	0.68
Access to information 1	0.70	0.66
Access to information 2	0.79	0.76
Access to information 3	0.61	0.68
Authorities Legitimacy 1	0.84	0.91
Authorities Legitimacy 2	0.88	0.89
Authorities Legitimacy 3	0.63	0.65
Perceived threat 1	0.77	0.73
Perceived threat 2	0.86	0.90
Perceived threat 3	0.22	0.23
Perceived threat 4	0.80	0.83
Perceived threat 5	0.76	0.76
Social pressure 1	0.78	0.71
Social pressure 2	0.57	0.60
Social pressure 3	0.52	0.58
Social pressure 4	0.56	0.70
Social pressure 5	0.61	0.51
Social pressure 6	0.56	0.38
Self-care 1	0.22	0.38
Self-care 2	0.37	0.44
Self-care 3	0.33	0.41
Self-care 4	0.52	0.58
Self-care 5	0.60	0.61
Self-care 6	0.66	0.58
Precautionary behavior 1	0.40	0.51
Precautionary behavior 2	0.60	0.64
Precautionary behavior 3	0.57	0.50
Precautionary behavior 4	0.28	0.33
Precautionary behavior 5	0.52	0.44
Precautionary behavior 6	0.41	0.46
Precautionary behavior 7	0.46	0.46
Wellbeing 1	0.77	0.79
Wellbeing 2	0.73	0.73
Wellbeing 3	0.78	0.72
Wellbeing 4	0.85	0.79
Wellbeing 5	0.60	0.57
Wellbeing 6	0.61	0.58
Physical Infrastructure on Resources	0.46	0.47
Family support on Resources	0.65	0.55
Social support on Resources	0.59	0.55
Access to information on Enablers	0.47	0.50
Authorities legitimacy on Enablers	0.47	0.50
Perceived threat on Challenges	0.42	0.47
Social Pressure on Challenges	0.38	0.45
Self-care on Responsible Behavior	0.75	0.66
Precautionary beh. on Responsible beh.	0.73	0.63
Resources on Positive environment	0.78	0.77
Enablers on Positive environment	0.22	0.29
Challenges on Positive environment	−0.49	−0.39

**FIGURE 2:**
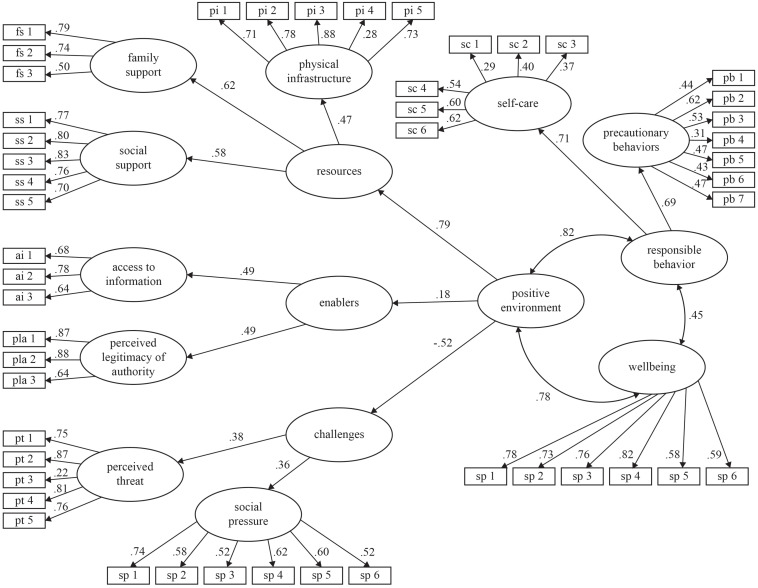
Test of the model of positive environments and precautionary behaviors against COVID-19. Goodness of fit: Satorra–Bentler χ^2^ = 2288.65 (1,114 df), *p* < 0.0001; BNNFI = 0.90, CFI = 0.90; RMSEA = 0.04.

Figure 2 shows the results of the structural equation model. All observed indicators (i.e., items) loaded significantly (0.05) on their corresponding first-order factors, indicating convergent construct validity for the scales. “Resources” coherently emerged from its first-order latent variables (physical infrastructure, λ = 0.47; family support, λ = 0.62; social support, λ = 0.58). The indicators of the “Environmental Enablers” factor (access to information, λ = 0.49; legitimacy of authorities, λ = 0.49) also loaded saliently and significantly on its second-order latent variable; as well as the indicators of “Environmental Challenges” (perceived threat, λ = 0.38; social pressure, λ = 0.36). In turn, those second-order factors made up the higher-order “Positive Environment” factor (Resources, λ = 0.79; Enablers, λ = 0.18; Challenges, λ = −0.52). A similar pattern was detected in the relationship between “Responsible Behavior” and its indicators (Self-care, λ = 0.71; Precautionary behavior, λ = 0.69). The lambdas between “Hedonic Wellbeing” and its observed indicators ranged from 0.59 to 0.82). As in the partial-data models, high and significant covariances between PE, Responsible Behavior, and Hedonic Wellbeing (0.82, 0.45, and 0.78) were obtained. The goodness of fit indicators [χ^2^ = 2288.65 (1,114 df), *p* < 0.0001, relative χ^2^ = 2.05; *BNNFI* = 0.90, *CFI* = 0.90; *RMSEA* = 0.04] reveal that the model is supported by the data. Although the *p* value associated to χ^2^ resulted significant (due to the large sample size), the rest of the goodness of fit indicators were appropriate.

## Discussion

This study investigated precautionary behaviors in relation to COVID-19 in the framework of environmental positivity. The results suggest that a PE can be characterized by the availability of physical and social resources, the existence of challenges, and the presence of environmental enablers of sustainable behavior (Corral-Verdugo et al., 2017). A PE, in turn, influences and is influenced by the responsible behaviors of individuals (self-care and precautionary actions that mitigate COVID-19) and the hedonic wellbeing they experience. According to the tested theory, a PE requires the presence of significant relationships between those indicators: they appear together when the environment is positive (Corral-Verdugo and Frías-Armenta, 2016). These features (resources, enablers, challenges, and their interrelation) make PEs sustainable environments. In addition, every element in the model produced favorable and not dissimilar (from each other) scores, which is required to demonstrate that a PE is present. Although variability was noticed in those scores, no significant discrepancies were found among the factors’ scores, with most of their means between 4.4 and 3.2 (1–5 range of response). The exceptions were legitimacy of authorities (2.9) and social pressure (2.5). Therefore, in general, it can be said that not only a correlation exists between all the factors, but also that their scores are of similar magnitude. These results are reinforced by the Cronbach’s alpha of the whole model (not previously reported in the previous versions of the paper) which resulted = 0.77, indicating internal consistency in the model (i.e., the factors contribute to the PE construct in an evenly way).

An important take away from these results is that the design and maintenance of PEs may represent a viable strategy for facing serious issues like the COVID-19 pandemic, while maintaining individual and group wellbeing. These concepts may further inform official response to other environmental challenges moving forward, like climate change.

The scales used to assess the variables of interest demonstrated internal consistency reliability (as assessed by Cronbach’s alpha) as well as convergent construct validity. The elevated degree of threat perception is understandable considering the significant losses associated with the pandemic in Mexico. The high rate of infection, death toll, as well as employment and economic losses have put strain on the population to a degree not witnessed in recent memory. Precautionary behaviors also produced high response levels, indicating concern for the propagation of the disease and individual efforts to mitigate the spread of infection. Participants reported high degrees of family and social support, resources that may help counteract the threat of COVID-19.

Scales assessing access to adequate physical infrastructure (areas to do physical activity, outdoor natural areas, household outdoor facilities, etc.), access to accurate information regarding COVID-19, and hedonic wellbeing produced moderate means. Green urban infrastructure and maintenance in many Mexican cities is lacking or unequally distributed (Fernández-Álvarez, 2017), which may explain the relatively low means for these measures. Individual means of hedonic wellbeing were also moderate, somewhat unusual for a Mexican sample, which normally report high wellbeing (Dugain and Olaberría, 2015). This phenomenon is not totally unexpected as high degrees of wellbeing in the context of an epidemic or pandemic health crisis may not be typical. Yang and Ma (2020) found the coronavirus epidemic led to a 74% drop in overall emotional wellbeing among Chinese participants shortly after official announcement of the outbreak. Considering the expectation that pandemic events will likely reduce individual and collective wellbeing, it is critical to take environmental measures into account. The model demonstrated that variations in wellbeing were associated with the degree to which participants reported environmental positivity (i.e., the more positive the environment, the greater the individual self-report wellbeing).

Participants reported low perception of authority legitimacy in handling the COVID-19 crisis as well as low social pressure to not engage in precautionary behaviors. Legitimacy of authority is traditionally perceived as low in previous studies including Mexican participants in a wide range of public aspects (Edmonds-Poli and Shirk, 2020), which may explain relatively low reports in this study. Perhaps promisingly, social pressure to not engage in precautionary behaviors was also relatively low, implying participants considered the seriousness of the outbreak or the importance of “flattening the curve” of new infection.

Our findings confirm previous reports of a positive relationship between sustainable (responsible) behaviors and psychological wellbeing (Prati et al., 2017). In this study, responsible (self-care, precautionary) behaviors moderately, positively and significantly correlate with hedonic wellbeing. This seems to imply that, in a PE, individuals who engage in behaviors intended to protect themselves and others (against COVID-19) may experience hedonic wellbeing, regardless of the inconvenience those practices imply. Of course, wellbeing can also be experienced as a consequence of prosocial and pro-environmental behavior outside a PE.

The “Resources” factor loaded four times higher than “Enablers” on environmental positivity. Neither legitimacy of authorities nor access to information contributed to environmental positivity as much as resources available to the individual (particularly social resources). Yet, studies have suggested that Mexican respondents, across different socioeconomic status, tend to engage in acts of solidarity not only directed toward family members and friends but also toward the population-at-large, even in the presence of low levels of enablers. These expressions of solidarity appear to occur with a similar intensity across nations, as the report by Butcher et al. (2010). Feelings of solidarity among individuals may likewise be influences by relatively low degrees of perceived legitimacy of Mexican authority, thus contributing to a sense of environmental positivity outside of an official sector context (Edmonds-Poli and Shirk, 2020) and practices of reading/accessing information among broad segments of the Mexican population (Kalman and Reyes, 2016). Our results indicate that these and other enablers of environmental positivity must be enhanced to increase both the level of participation in responsible actions and individual wellbeing.

This study is not without limitations. Firstly, due the conditions of physical distancing imposed by the pandemic, obtaining a representative sample was difficult. The sampling technique used may have caused a biased sample, where middle-class and more educated individuals were over-represented. Moreover, several participants did not respond to all the items. Over 200 participants, whose data were not completely available, were left out the analyses, so that the structural models were affected by these losses since the analysis does not allow for a single missing data. Secondly, the model investigated some indicators of environmental positivity during the COVID-19 pandemic but there are certainly other factors that may be of importance. Future studies could include the role played by tangible resources such as access to food, household habitability, and financial resources. Likewise, only information and legitimacy of authority were assessed as enabler and it has been shown that community capacity [see Hartley and Jarvis (2020)] has an important role in the absence of legitimacy of authority. Thirdly, this model presents a high level of complexity because the explanation of complex phenomena is usually not better served by simpler models. Sustainable environments and behaviors are a case of this type of complex phenomena. Therefore, model estimation results more difficult and goodness of fit may be affected (Gribbons and Hocevar, 1998), which made us to drop three items from one scale.

Finally, since the data was collected through self-report questionnaires, some responses may be influenced by social desirability. In addition, part of the interrelations between the indicators of the PE may be due to the common method variance. Despite these limitations, this study provides clues for the design of PEs that help individuals cope during crisis. The present research likewise adds to our knowledge of the situational and psychological factors associated with precautionary practices against COVID-19.

### Recommendations

Our results suggest that new policies should include efforts to promote PEs by establishing and fostering resources and enablers that may help counter the challenges faced by individuals, particularly as it relates to crisis events like the COVID-19 pandemic. Likewise, we suggest that resources that stimulate social networks (family or community) and provide access to safe outdoor spaces are important for creating PEs. Although social distancing has hindered access to the usual sources of sociality, leading to social isolation and subsequent psychopathology (Usher et al., 2020), the practice represents the first line of defense against the continued spread of COVID-19. Previous literature has shown the importance of social support in mental health recovery after widespread disasters such as Hurricane Katrina (Chan et al., 2015). Despite physical distancing measures, modern technology solutions brought about by widespread access to the internet may help buffer loneliness and isolation by providing a way to provide and receive social support (Saltzman et al., 2020). Online “cocktail” parties, social group chats, keeping up with of friends through social media are only a few ways individuals have attempted to reclaim normalcy in the face of the pandemic. A recent study suggested that before COVID-19, students facing a strong academic stressor had buffering effects from online but not face-to-face feedback (Rodríguez-Hidalgo et al., 2020). The interaction between physical and social qualities of PEs and its influence on precautionary behaviors and wellbeing should also be considered. Previous research by Evans et al. (1996), and Corral-Verdugo et al. (2015a), for instance, has shown that the physical environment of homes affects mutual support and stress of family members. Results of the present study reinforces that evidence, stressing the importance of habitability of households and its effect on family wellbeing.

Access to safe outdoor spaces during periods of physical distancing is critical, particularly considering the rise in rates of self-report loneliness, anxiety, and depression (Pouso et al., 2020). Access to these spaces is important, in part because outdoors is possible to meet people (keeping physical distance), and because parks and streets with green areas are restorative. Many areas have begun to incorporate nature to workplace environments to increase wellbeing during the COVID-19 era. For instance, former triage tents at Mount Sinai Hospital have been converted into “recharge rooms” for healthcare workers on the frontlines of the COVID-19 response (Naomi, 2020). The “nature-filled settings” include “immersive nature environments with music, scent, lighting, and sound” (Abilities Research Center, 2020). Recent reports have also made long and short-term recommendations to improve access to safe outdoor environments, such as adopting open or slow street initiatives and creating built environments for all users (Slater et al., 2020). It is important to note that policies similar to these, which provide safe access to outdoor areas be incorporated to different living areas and workplaces.

Environmental enablers are likewise critical components of an effective response to adversity. Our results suggest official action should be directed toward providing clear information, data, and evidence of the actions to be taken to prevent further COVID-19 spread, and how those actions, in turn, protect people and foster PEs. Confidence or perceived legitimacy of authority appears deficient at this moment among the sampled population. Trust in authority may serve to encourage social adherence to the guidelines suggested by public officials. Focus should be paid to identifying and addressing deficiencies in perceived trust to promote confidence among all involved parties. However, even in situations of low levels of public trust and political legitimacy nations have been able to contain spread through community capacity (Hartley and Jarvis, 2020). Hong Kong, while in political turmoil, has effectively mitigated the spread of COVID-19. Authors attribute this to community initiative in the absence of widely accepted policy posture. Research in Australia showed that trust in authorities or fear of legal sanctions did not predict compliance (Murphy et al., 2020). These studies generally suggest that public policies need to focus on persuading citizens that everyone has a duty to protect those most vulnerable to the disease. The results of this study support this notion, particularly when considering higher response rates for concern about others, as opposed to concern about one’s self in the context of COVID-19. Promoting responsible behavior is critical for the development of positive socio-physical environments, which will in turn increase wellbeing, and result chain reaction of positive coping strategies in the face of crisis.

Given that people, in times of crisis, turn to their leaders for credible scientific information and related guidance, it is pivotal that those voices represent the best information aimed at the best solutions to tackle difficult issues. Technology (the internet, social media) must be leveraged to raise awareness of the critical importance of self-care and use of techniques that promote wellbeing and PEs. The negative societal effects of the COVID-19 pandemic reach beyond the scope heath and behavioral health care. As such, official response to the threat of the pandemic must consider the depth and breadth of the challenges, resources, and enablers that promote PEs and individual wellbeing.

## Data Availability Statement

The raw data supporting the conclusions of this article will be made available by the authors, without undue reservation.

## Ethics Statement

The studies involving human participants were reviewed and approved by Comité de Ética en Investigación de la Universidad de Sonora. The patients/participants provided their written informed consent to participate in this study.

## Author Contributions

VC-V and NC-F contributed by writing, reviewing, and editing. EP-T ran formal analysis and organized databases. VC-V contributed by supervising this study and its methodological tasks (methodology) were designed by VC-V, MF-A, and NC-F. VC-V and ML provided the writing of the original draft. All authors contributed with conceptualization, design of this study, manuscript revision, and read and approved the submitted version.

## Conflict of Interest

The authors declare that the research was conducted in the absence of any commercial or financial relationships that could be construed as a potential conflict of interest.
